# Ultra-compact and high-performance polarization beam splitter assisted by slotted waveguide subwavelength gratings

**DOI:** 10.1038/s41598-020-69749-7

**Published:** 2020-07-30

**Authors:** Chia-Chih Huang, Chia-Chien Huang

**Affiliations:** 1 Department of Electronic Engineering, Tungnan University, No. 152, Sec. 3, Beishen Rd., Shenkeng Dist., New Taipei City, 222 Taiwan, ROC; 2 Department of Physics and Institute of Nanoscience, National Chung Hsing University, 145, Xingda Rd., Taichung, 402 Taiwan, ROC

**Keywords:** Optics and photonics, Physics

## Abstract

We propose an ultra-short polarization beam splitter (PBS) consisting of two slot waveguides assisted by slotted waveguide subwavelength gratings (SWSWGs), located between the two slotted waveguides. By controlling the optical momentum of evanescent waves with the anisotropic characteristics of the SWSWGs, we considerably suppress and enhance the couplings of transverse-electric (TE) and transverse-magnetic (TM) modes, respectively, concurrently improving performances and reducing length of the proposed PBS, compared with conventional slotted waveguide couplers (CSWCs). Exceptionally, a transition point is found to show almost zero crosstalk between waveguides for the TE mode, i.e., infinite coupling length. Differing from conventional single-material SWGs, the SWSWGs not only simplify the fabrication process but improve polarization extinction ratio (PER). Numerical results demonstrate the improvement in PER_TM_ (PER_TE_) from approximately 13 (23) dB for the CSWCs to 26 (24) dB for the present structure, with a > 70% reduction in device length, operating at the wavelength of λ = 1,550 nm. Our design achieves performance of PER_TM_ > 25 dB and PER_TE_ > 20 dB, and insertion loss (IL) < 0.05 dB for TE and < 0.3 dB for TM modes within a bandwidth width (BW) of ~ 50 nm from λ = 1,530 to 1,580 nm. Additionally, geometry deviation is also investigated to assess experimental tolerance. The present idea provides an approach for improving PER, device length, and operating BW of PBSs composed of various waveguide couplers.

## Introduction

Combing electronic and photonic devices in photonic integrated circuits (PICs) is essential to progress in high-density integration and nanophotonics. Silicon-on-insulator (SOI) technology is the candidate platform for realizing this long-term goal^1,2^ as it offers two main benefits: Mature complementary metal-oxide semiconductor (CMOS)-compatible technology, and high refractive index contrast, enabling more compact devices. Inevitably, the SOI platform causes strong polarization dependence due to its high birefringence, which is detrimental in optical-fiber systems. Therefore, use of polarization-division multiplexing devices including polarization beam splitters (PBSs) and rotators has been reported to deal with the issue^3–5^. The most widely used are PBSs designed to separate two orthogonal polarization states: The transverse-electric (TE) mode the and transverse-magnetic (TM) mode. As a result, PBSs employing different splitting approaches^5,6^ have been proposed, such as adiabatic taper waveguides (ATWs)^6^, multi-mode interference (MMI)^7,8^, waveguide grating couplers^9,10^, computationally optimized metamaterials^11^, and directional couplers (DC)^12–15^. Generally, there are some criteria including: (1) polarization extinction ratio (PER); (2) insertion loss (IL); (3) operating bandwidth (BW); (4) the device dimension, and (5) fabrication difficulty to evaluate the merits of a PBS. Although the ATW-based PBS^6^ of hundreds of micrometers is long due to its gradually evolving geometry, it offers high fabrication error tolerance and broadband operation. MMI-based PBSs^7,8^ require a simpler fabrication process due to their use of wide rectangular waveguides but have the drawback of an extremely long structure (> 1,000 μm) unless assisted design is used. Waveguide grating-based PBSs^9,10^ can achieve a footprint of tens of micrometers but their complex fabrication and large scattering loss make them be often used in specific condition such as coupling power from one component to another. Recently, researchers have adopted free-form metamaterials to design PBSs with an ultra-small area of 2.4 × 2.4 μm^2^ using computational optimization^11^. Nevertheless, the time-consuming design and a low PER of ~ 10 dB with a narrow BW of 32 nm make it unsatisfactory in designing high-performance PBSs. Compared with these mechanisms mentioned above, DC-based PBSs^12–15^ are more attractive due to their comparatively small dimensions, acceptable performances, various design approaches, and simpler structures.

For the DC-based PBSs, the phase matching condition (PMC) is satisfied to separate two polarization modes, in which one mode is coupled to the cross channel and the other mode propagating along the through channel is designed to be deviated from the PMC. Hence, DC-based PBSs can be built flexibly by using several possible waveguide structures. In Ref.^12^ reported by Fukuda et al., a DC-based PBS with a footprint of 7 × 16 μm^2^, implemented with a Si-strip coupler on an SOI platform, with calculated PER_TE_ (PER_TM_) about 15 (10) and IL_TE_ (IL_TM_) about 0.5 (0.5) dB, in the C-band range. In Ref.^13^, Guan et al. proposed an asymmetric DC-based PBS composed a silicon (Si) strip and a hybrid plasmonic waveguide. Within a 120 nm working BW, the footprint of the device is with 1.9 × 3.7 μm^2^ and their PERs are > 12 dB. Although that device length is extremely short, the PERs of 12 dB require considerable improvement. Instead of adopting two Si strips, Yue et al.^14^ used two slot waveguides^16,17^ to build a PBS to enhance polarization dependence by effectively increasing TM mode coupling compared with that of Ref.^14^. The device length of Ref.^14^ is thus shrunk to 46.7 μm compared with that of 350 μm using two Si strips^12^. However, the calculated PERs of Ref.^14^ were around 20 dB within an 18 nm operating BW. Subsequently, Zhang et al.^15^ experimentally demonstrated the PERs of 16.8 and 14.1 dB for TE and TM modes, respectively, for the design of Ref.^14^.

In principle, subwavelength gratings (SWGs), comprising dielectric strips of much smaller dimension than the working wavelength, which behave as homogeneous media with an equivalent anisotropic refractive index^23^ depending on the geometry of the structure and the polarization of the electromagnetic wave propagating within it, alleviating the limited choice of material refractive indices and further enabling the design of high-performance photonic devices. The desired material properties can be controlled by varying the constituent dielectrics, duty cycle, or number of gratings, providing an extra degree of freedom for tailoring the required mode characteristics. Many photonic devices^18–25^ designed by adopting SWGs in the waveguides have recently been reported, following the modern fabrication technology. More recently, Jahani and Jacob^26–28^ located SWGs in the regions between waveguides to significantly reduce crosstalk. Also, Xu et al.^29^ adopted SWGs in both the waveguide and cladding regions to form a hetero-anisotropic slab structure, while the slab performs as an MMI coupler and a two isolated waveguides for the TM and TE polarizations, respectively. Li et al.^30^ introduced a pair of cascaded dual-core adiabatic tapers consisting of a tapered SWG and regular adiabatic tapered waveguides to achieve low ILs and high PERs. In the present work, we propose a PBS comprising two main slotted waveguides, assisted by slotted waveguide subwavelength gratings (SWSWGs) located between the two slotted waveguides. By controlling the optical momentum of evanescent waves with the anisotropic SWSWGs, we can not only significantly suppress the crosstalk of TE mode but considerably enhance the coupling strength of TM mode, to substantially improve PER_TM_ and reduce the device length by around a quarter compared with a conventional slotted waveguide coupler (CSWC)^14^. In addition, a transition point showing almost zero crosstalk (i.e., infinite coupling length) of the TE mode is found in the present structure.

## Results and discussion

### Analysis of mode coupling based on optical momentum of evanescent wave

**Figure 1:**
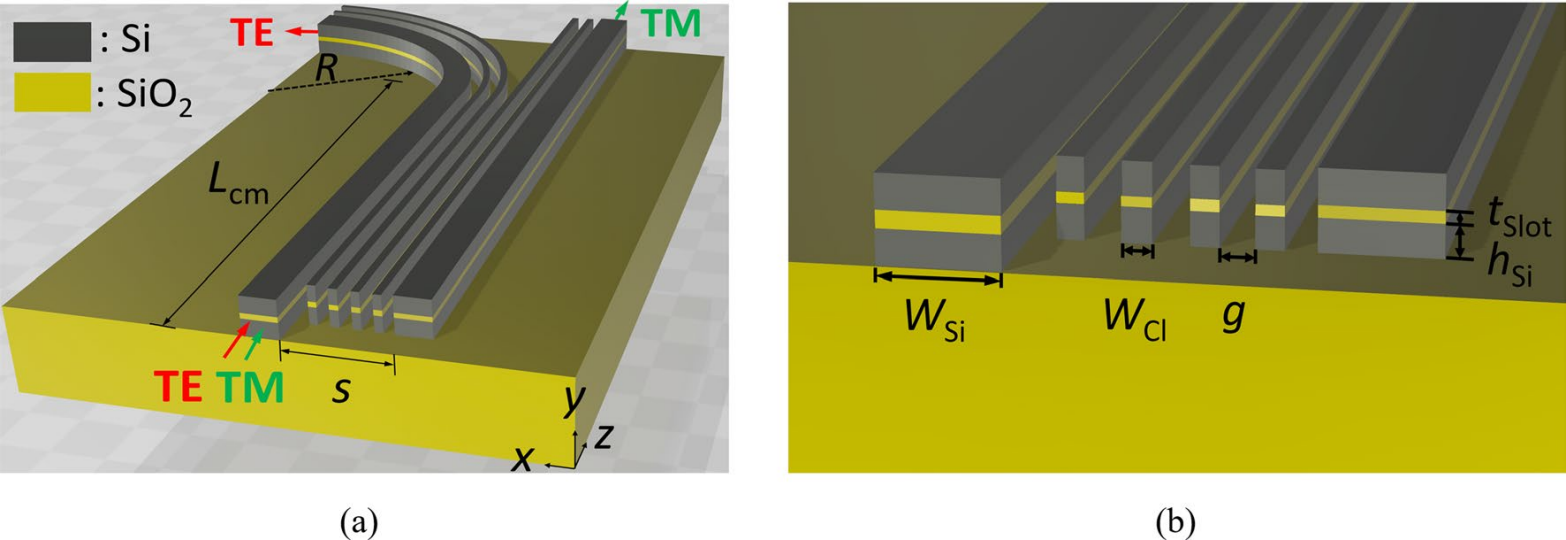
(**a**) 3D schematic diagram of our designed PBS and (**b**) the zoomed-in view of the input port.

A 3D schematic of the proposed PBS is shown in Fig. 1a, and the zoomed-in view of the cross section of the input is shown in Fig. 1b. The proposed design comprises two horizontal slotted waveguides with SWSWGs located between the slotted waveguides. The two slotted waveguides and SWSWGs all comprise a low-index SiO_2_ slot layer sandwiched between two high-index Si layers. To effectively decouple the two output powers, a 90° angled slotted waveguide with the radius of curvature, *R*, is connected to a slotted waveguide delivering the TE mode, while another straight slotted waveguide carries the power of the TM mode. Note that an equal number of SWSWGs is distributed between each of the two output ports. The substrate and cladding are SiO_2_ and air, respectively.

**Figure 2:**
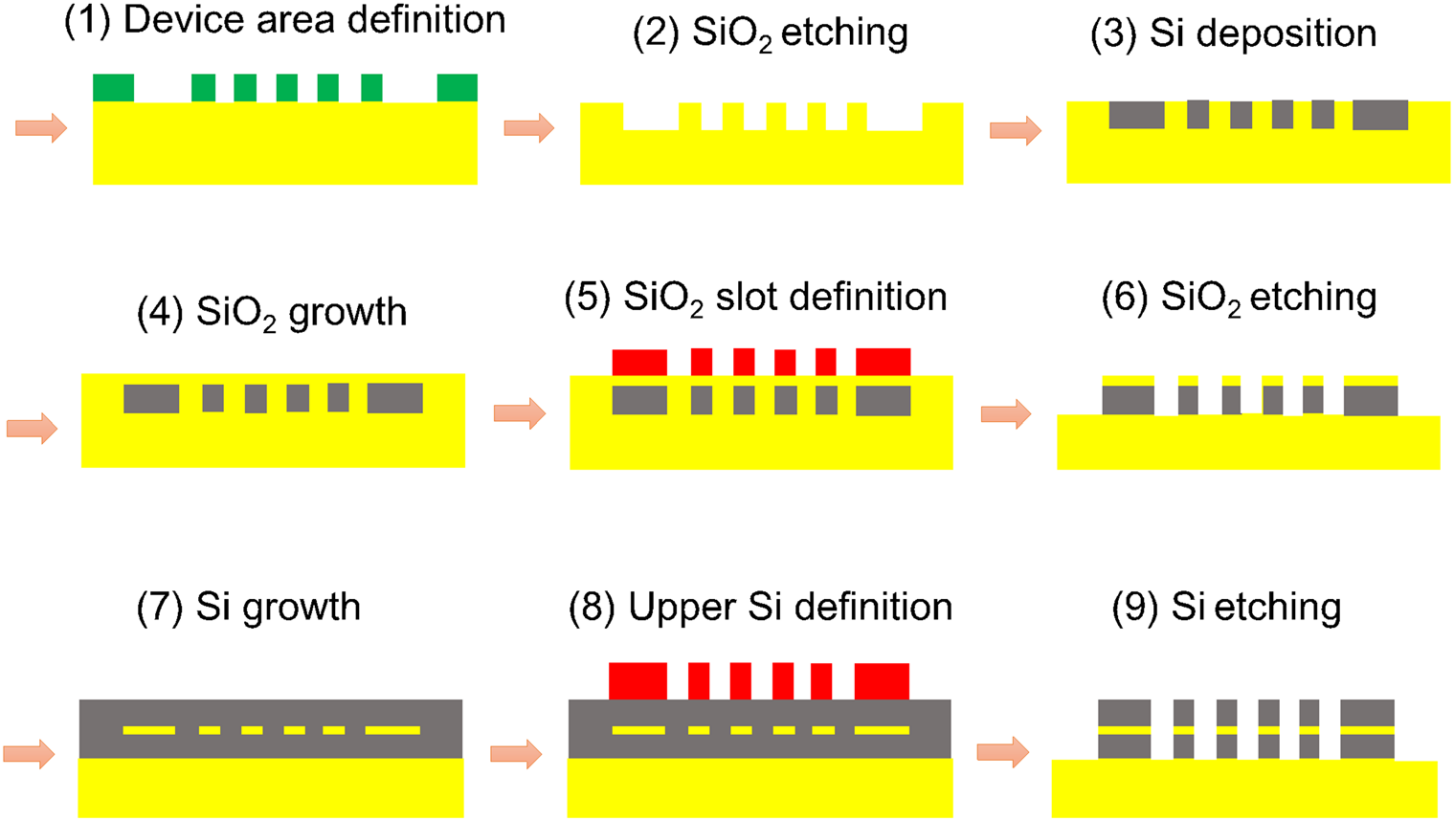
Schematic diagram of the fabrication processes of the present device.

The relevant parameters are the Si width of the slotted waveguides *W*_Si_ with edge-to-edge spacing *s*, the width of the SWSWGs *W*_cl_, and the height *h*_Si_ and thickness *t*_slot_ of the Si and slot layer. The SWSWG pitch is set to *Λ* = *W*_cl_ + *g* and with duty cycle *ρ* = *W*_cl_/*Λ*, where *g* is the gap between the strips. Note that *Λ* is set under the subwavelength to suppress diffraction effects^23^. The input TM mode with major electric component in the *y*-direction, *E*_*y*_, is coupled to the adjacent slotted waveguide with the help of the SWSWGs, while the TE mode with major electric component in the *x*-direction, *E*_*x*_, is guided along the through bar connected by a curved waveguide with negligible power coupling. The processes required for practical fabrication of the proposed device are shown schematically in Fig. 2. Prior to these processes, the patterned hard masks for the curved TE channel, straight TM channel, and SWSWGs are fabricated using high resolution electron beam lithography. After that, the fabrication processes of the proposed structure are shown below:

(1) Preparing a SiO_2_ substrate (yellow) for deposition with a negative photoresist (PR) film (green) of height *h*_Si_ to pattern the lower Si layer of the proposed PBS with the preceding masks, a PR exposure with ultraviolet (UV) light, development, and an etching process. (2) The pattern of the proposed structure is formed by etching SiO_2_ and lifting off the PR film. (3) After depositing a Si layer in the trenches using chemical vapor deposition in the wells, a chemical mechanical polishing (CMP) process is used to attain a flat plane. (4) Similarly, a SiO_2_ layer of height *t*_slot_ is deposited using thermal oxidation, then CMP is used to obtain a flat SiO_2_ surface. (5) To define a SiO_2_ layer, a positive PR film (red) is deposited on the flat SiO_2_ surface. The patterned hard masks are used to pattern the PR film. (6) Before lifting off the PR, reactive ion etching is used to form the SiO_2_ slot layer. (7) Depositing a Si layer with the height *h*_*Si*_, and using CMP process to have a flat Si film. (8) The upper Si layer is defined using the same process as (5) except for deposition of a positive PR film on the Si layer. (9) After Si etching, the PR film is removed to obtain the proposed device. Note that the fabrication processes of the proposed structure are similar to those of a CSWC, making fabrication of the proposed PBS comparatively simple.

**Figure 3:**
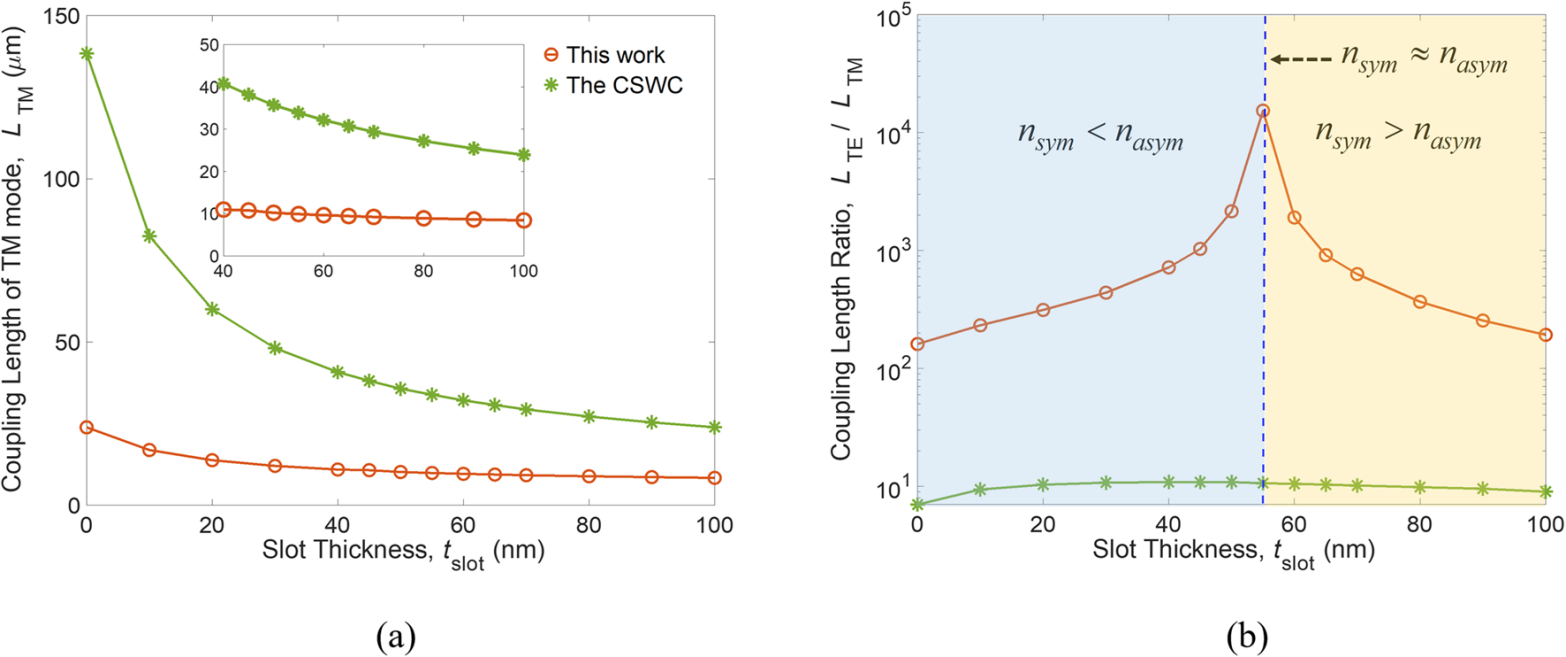
(**a**) Coupling lengths of TM (*L*_TM_) and TE (*L*_TE_) modes, and (**b**) the coupling-length ratio *L*_TE_/*L*_TM_ as a function of slot thickness *t*_slot_ for the CSWC and the present design.

For different polarizations, the SWG offers an extra degree of freedom according to the effective-medium theory (EMT)^31^ to design the material anisotropy by adjusting its duty cycle, material constituents, and grating profile (see the “Methods” section). Besides, the coupling length of a coupled waveguide is determined by using the formula of *L*_*i*_ = *λ*/[2(*n*_*i, sym*_ – *n*_*i, aym*_)] in coupled mode theory^32^, where *i* is the TE or TM, and *n*_*i,sym*_ and *n*_*i,asym*_ are the effective indices of the symmetrical and anti-symmetrical modes of the *i* mode, respectively. Before addressing the propagation properties of the proposed PBS, we first analyze its mode characteristics and coupling strengths. In this work, COMSOL Multiphysics software employing a rigorous finite element method was used to calculate the simulation results. The refractive indices of Si and SiO_2_ at wavelength *λ* = 1,550 nm were *n*_*Si*_ = 3.480 and *n*_SiO2_ = 1.444^33^, respectively. The geometry parameters selected were as follows: *h*_Si_ = 150 nm; *W*_cl_ = 75 nm; *g* = 50 nm; *ρ* = 0.6; *W*_Si_ = 400 nm; and *s* = 550 nm. Figure 3a shows the coupling length of the TM mode (*L*_TM_) versus slot thickness, *t*_slot_, for the proposed and CSWC structures. We observe that *L*_TM_ dramatically reduces as *t*_slot_ increases from 0 to 60 nm. Further increasing *t*_slot_, the *L*_TM_ varies slightly. This is because a thicker *t*_slot_ leads to looser mode confinement, increasing the mode coupling strength. We know that the length of a PBS device is determined by the shorter mode coupling length. In the proposed structure, *L*_TM_ is much shorter than *L*_TE_. Therefore, the TM mode is designed to be coupled to the cross bar, while the TE mode propagates along the through bar. Our numerical results show that the values of *L*_TM_ at *t*_slot_ = 0 (i.e., two Si strips without a slot layer^12^) and 55 nm are around 137 and 34 μm, respectively, for the CSWC^14^. In contrast, the *L*_TM_ at *t*_slot_ = 55 nm for the proposed PBS is only 9.9 μm long; a 70% reduction in device length compared to the CSWC, making the footprint of the proposed PBS ultra-compact. To evaluate the PER and IL of the PBS, another essential index, *L*_TE_ / *L*_TM_, referred to as the coupling-length ratio of TE to TM modes, is shown in Fig. 3b.

A larger *L*_TE_ / *L*_TM_ means that less TE power couples to the cross channel (i.e., more TE power is preserved in the through channel), obtaining higher PER_TM_ and lower IL_TE_ (see the definitions of PER and IL in the “Methods” section). For the CSWC and our proposed structure, the maxima of *L*_TE_/*L*_TM_ are 10.6 and 15,336 (three orders of magnitude higher), respectively, at *t*_slot_ = 55 nm. Remarkably, a non-trivial regime where *n*_*sym*_ < *n*_*asym*_ for the TE mode (light yellow region in Fig. 3b) appears in our structure but not in the CSWC. Moving from a trivial coupling regime where *n*_*sym*_ > *n*_*asym*_ to a non-trivial coupling regime where *n*_*sym*_ < *n*_*asym*_, there is a transition point at *t*_slot_ $\approx$ 55 nm where *n*_*sym*_ $\approx$ *n*_*asym*_*,* such that the coupling length of the TE mode approaches infinity, i.e., TE mode crosstalk is almost completely suppressed. As a result, selecting *t*_slot_ = 55 nm can achieve lowest coupling of the TE mode to the cross bar, thus significantly improving PER_TM_.

**Figure 4:**
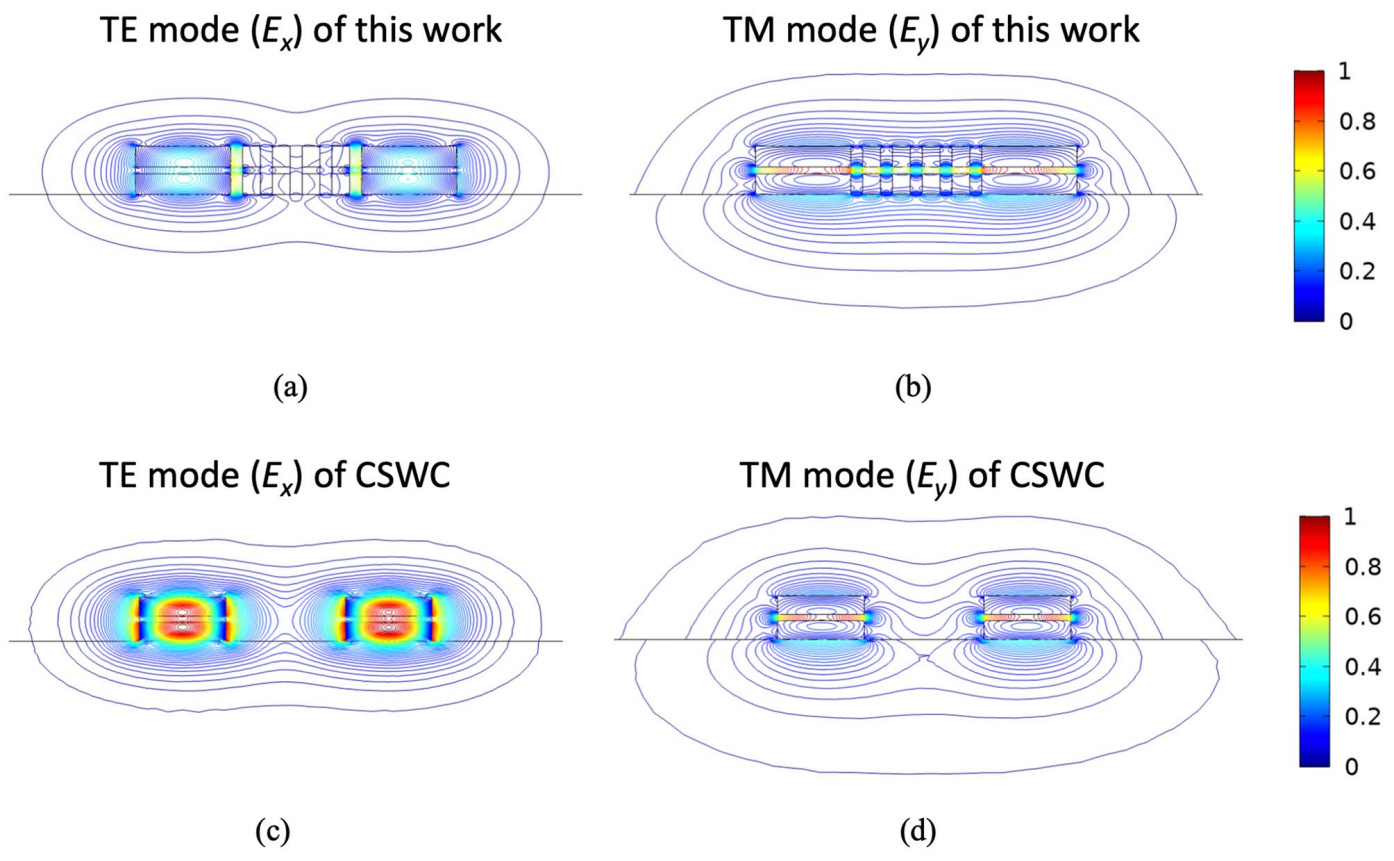
Field profiles of (**a**) TE and (**b**) TM symmetrical modes of the present design, and those of (**c**) TE and (**d**) TM ones of the CSWC^14^, for *t*_slot_ = 55 nm, *W*_Si_ = 400 nm, *h*_Si_ = 150 nm, *W*_cl_ = 75 nm, *g* = 50 nm, and *s* = 550 nm.

The exceptionally low waveguide crosstalk can be explained by the presence of the SWGs^26^. Theoretically, mode confinement is determined by the refractive index contrast of the core and cladding. Therefore, TE mode coupling strength should increase if we replace the air cladding in the CSWC with anisotropic SWGs. However, the results obtained are counterintuitive. This can be understood by that the decay rate of the evanescent wave of the TE mode, *k*_TE_, is determined by the ratio $\sqrt {\varepsilon_{z} /\varepsilon_{x} }$^26^, and the condition *ε*_*z*_ (= *ε*_*y*_) > *ε*_*x*_ is always fulfilled according to Eqs. (2) and (3). For an isotropic cladding (*ε*_*z*_ = *ε*_*x*_), *k*_TE_ is smaller than that of an anisotropic cladding, resulting in a longer evanescent tail for the TE mode. For the TM mode, the decay rate of its evanescent wave, *k*_TM_, depends on $\sqrt {\varepsilon_{z} /\varepsilon_{y} } = 1$, making the confinement of TM mode is not affected by the anisotropic SWSWGs but is determined by the averaged permittivity of the SWSWG structure. The large permittivity of the cladding leads to looser TM mode confinement, thus increasing coupling between the two slot waveguides. In addition, the underlying mechanism of the transition point can be attributed to the anisotropic cladding of the SWSWGs causing the coupling coefficient of the TE mode to approach zero. To demonstrate the above explanations, field contours with a normalized amplitude of from 0 to 1 for TE (*E*_*x*_) and TM (*E*_*y*_) symmetrical modes for the proposed SWSWGs are shown in Fig. 4a,b, respectively; those for the CSWC^14^ are shown in Fig. 4c,d, respectively. Evidently, the field overlap between the two main waveguides is significantly suppressed (enhanced) for the TE (TM) mode, compared with those of the CSWC.

### Propagation characteristics and geometry tolerance of the present device

**Figure 5:**
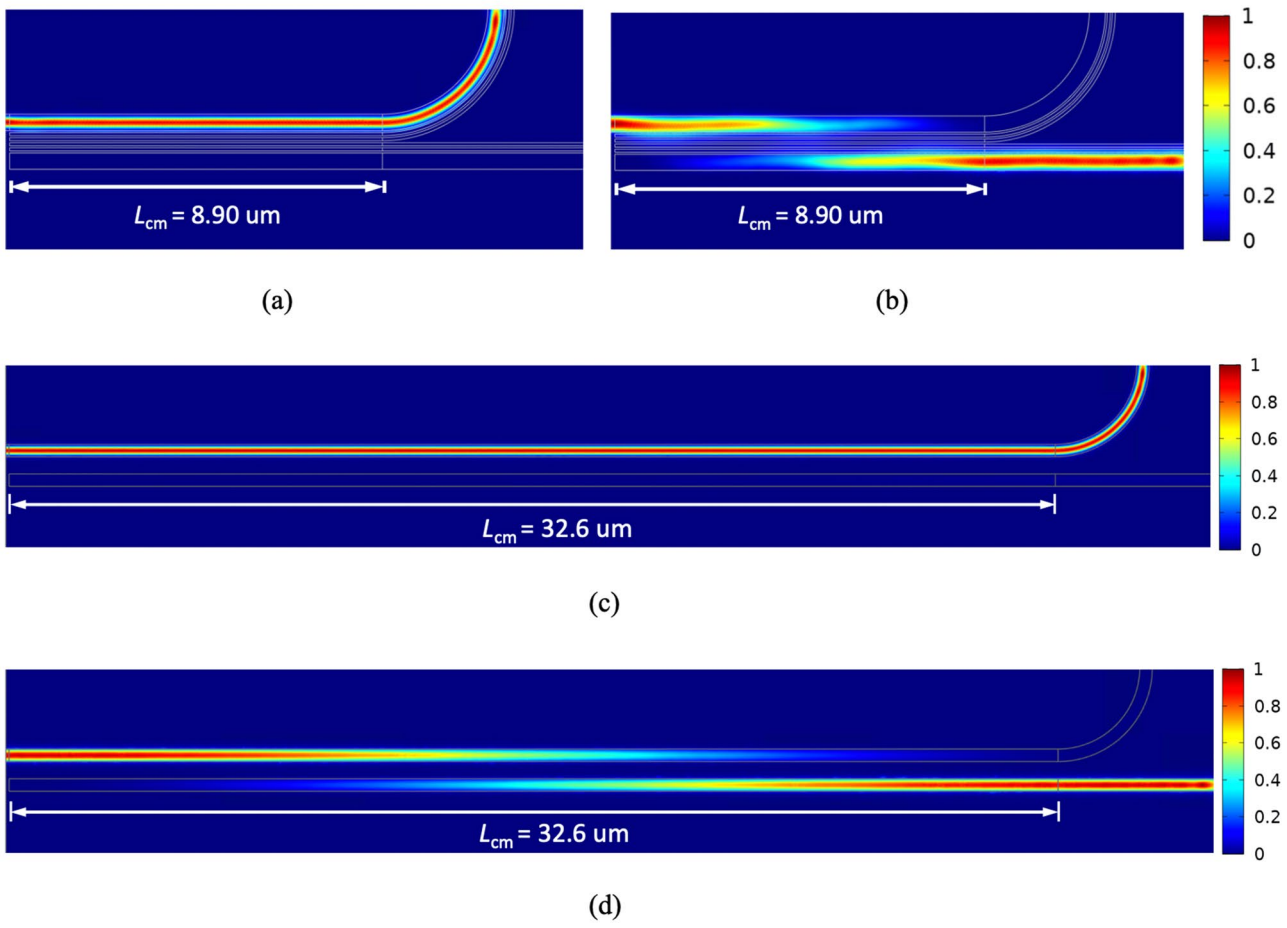
Poynting power evolutions for the (**a**) TE and (**b**) TM modes of the proposed PBS with *L*_TM_ = 9.91 μm (*L*_cm_ = 8.9 μm), and those of the (**c**) TE and (**d**) TM modes of the CSWC with *L*_TM_ = 33.88 μm (*L*_cm_ = 32.6 μm).

After obtaining the coupling length, we study the propagation characteristics of the proposed PBS. With the parameters used in Fig. 4 and *R* = 3 μm, Poynting power evolutions for the TE and TM modes are shown in Fig. 5a,b, respectively, at the modified coupling length, *L*_cm_ = 8.9 μm (*L*_TM_ = 9.9 μm), and those for the CSWC with *L*_cm_ = 32.2 μm (*L*_TM_ = 33.88 μm) are shown in Fig. 5c,d, respectively. Here, the *L*_cm_ is optimized for performance from the coupling length of TM mode, *L*_TM_. As shown in Fig. 1a, a bent waveguide is connected at the end of the through channel to decouple the two modes. We moderately shorten the *L*_TM_ to be *L*_cm_ because the coupling remains for a short distance around 1 μm within the bent waveguide. The proposed PBS achieves PER_TE_ = 24.13 dB and PER_TM_ = 26.02 dB, and IL_TE_ = 0.02 dB and IL_TM_ = 0.18 dB. In contrast, the CSWS achieves PER_TE_ = 23.13 dB and PER_TM_ = 13.55 dB, and IL_TE_ = 0.17 dB and IL_TM_ = 0.04 dB.

**Figure 6:**
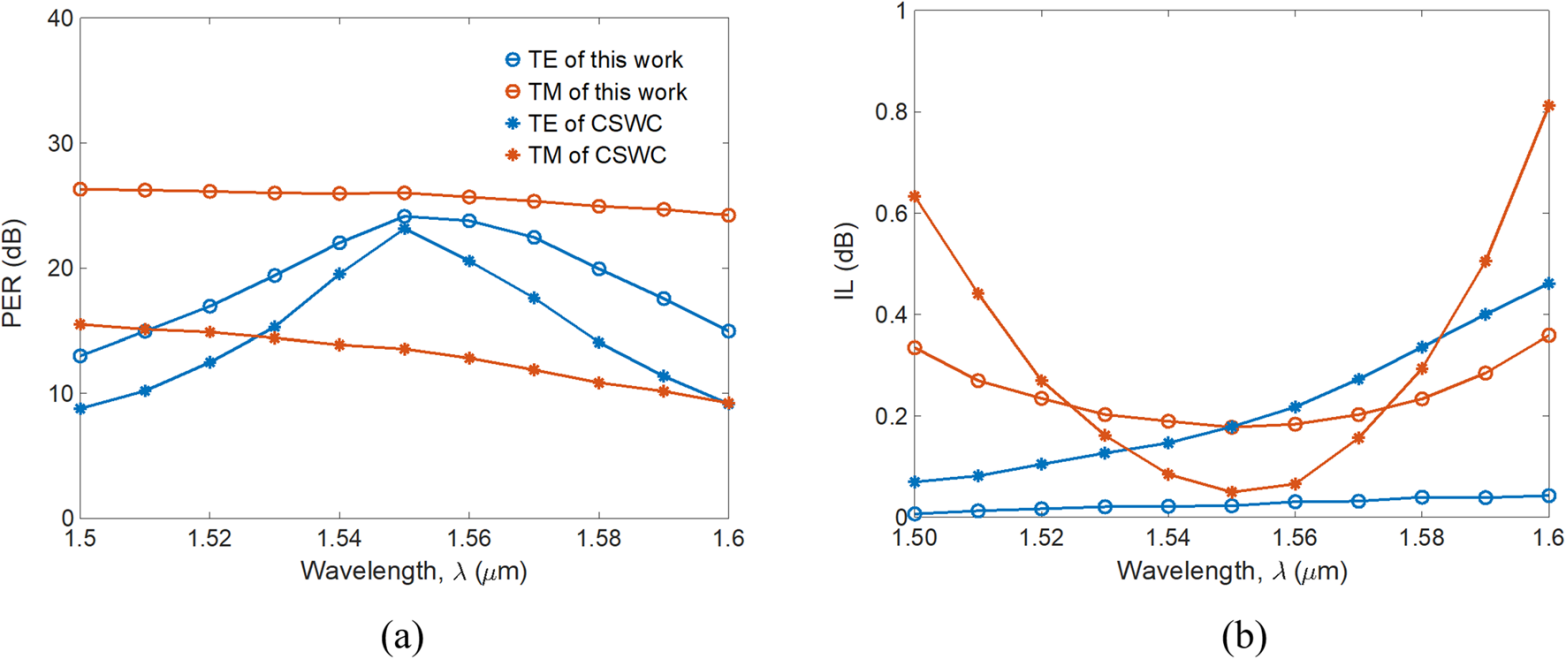
(**a**) PER and (**b**) IL as a function of wavelength with *t*_slot_ = 55 nm for the present structure and the CSWC.

Furthermore, PERs and ILs versus wavelength are shown in Fig. 6a,b, respectively, to evaluate the working BW of the PBS. We observe that PER_TE_ depends significantly on wavelength due to the short *L*_TM_. By contrast, PER_TM_ shows slight variation on wavelength due to the extremely long *L*_TE_ = 151.3 mm. Within a BW of ~ 100 nm from *λ* = 1,500 to 1,600 nm, the PER_TM_ (PER_TE_) of our device is greater than that of the CSWS by around 13 (3) dB. The proposed PBS achieves performance of PER_TM_ > 25 dB, PER_TE_ > 20 dB, IL_TE_ < 0.05 dB, and IL_TM_ < 0.3 dB within a BW of ~ 50 nm from *λ* = 1,530 to 1,580 nm. However, the PER_TM_ of the CSWC is less than 15 dB in the BW from *λ* = 1,500 to 1,600 nm. In fact, the IL_TE_ < 0.05 dB of the proposed structure extends to the entire band of 100 nm due to the transition point of *t*_slot_ = 55 nm being selected.

**Figure 7:**
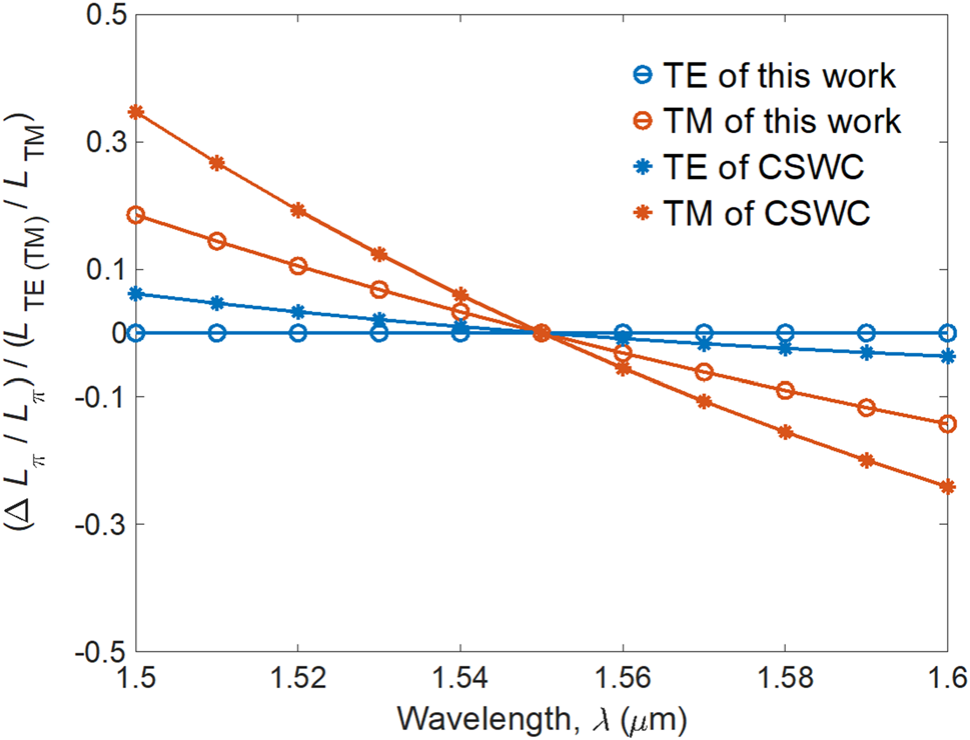
Wavelength sensitivity (Δ*L*_π_/*L*_π_)/(*L*_TE(TM)_/*L*_TM_) as a function of wavelength for the present structure and the CSWC.

Theoretically, SWG structure exerts different effects on the propagation constants of symmetric and asymmetric modes with different field distributions, making the mode dispersion can be tailored by adjusting the geometry of SWG structure to reduce the wavelength sensitivity^23^. To demonstrate the expansion of the operating bandwidth of the present design, the relative variation of coupling length normalized by the coupling length ratio (i.e., (Δ*L*_π_/*L*_π_)/(*L*_TE(TM)_/*L*_TM_) reflecting wavelength sensitivity) versus the wavelength is shown in Fig. 7. The reason of normalizing Δ*L*_π_/*L*_π_ by the coupling length ratio is that the performances (see Fig. 6) are computed at the *L*_TM_ (i.e., device length) of *λ* = 1.55 μm. Therefore, Δ*L*_π_ /*L*_π_ reflects the wavelength sensitivity only for the TM mode (the coupling length ratio chosen here is *L*_TM_/*L*_TM_ = 1) not for the TE mode (the coupling length ratio is *L*_TE_/*L*_TM_). The extremely slight wavelength sensitivity of the TE mode of this work demonstrates the high PER_TM_ as shown in Fig. 6. By contrast, the wavelength sensitivity of the TM mode shows moderate variation of PER_TE_. We observe that the wavelength sensitivities of TE and TM modes of this work are smaller than those of the CSWC from *λ* = 1.5 to 1.6 μm, confirming the larger operation bandwidth than that of conventional DC-based PBSs.

**Figure 8:**
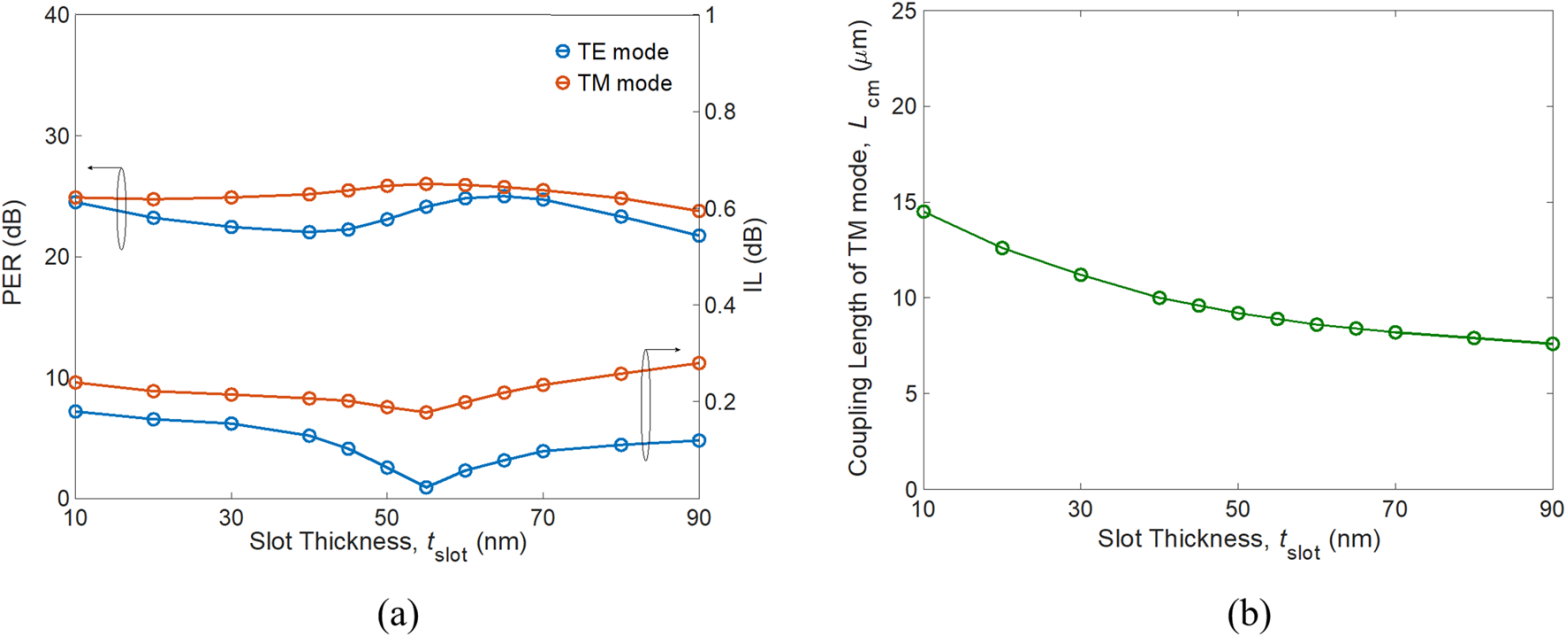
(**a**) PER (left axis) and IL (right axis), and (**b**) modified coupling length of the TM mode *L*_cm_ as a function of slot thickness *t*_slot_.

**Figure 9:**
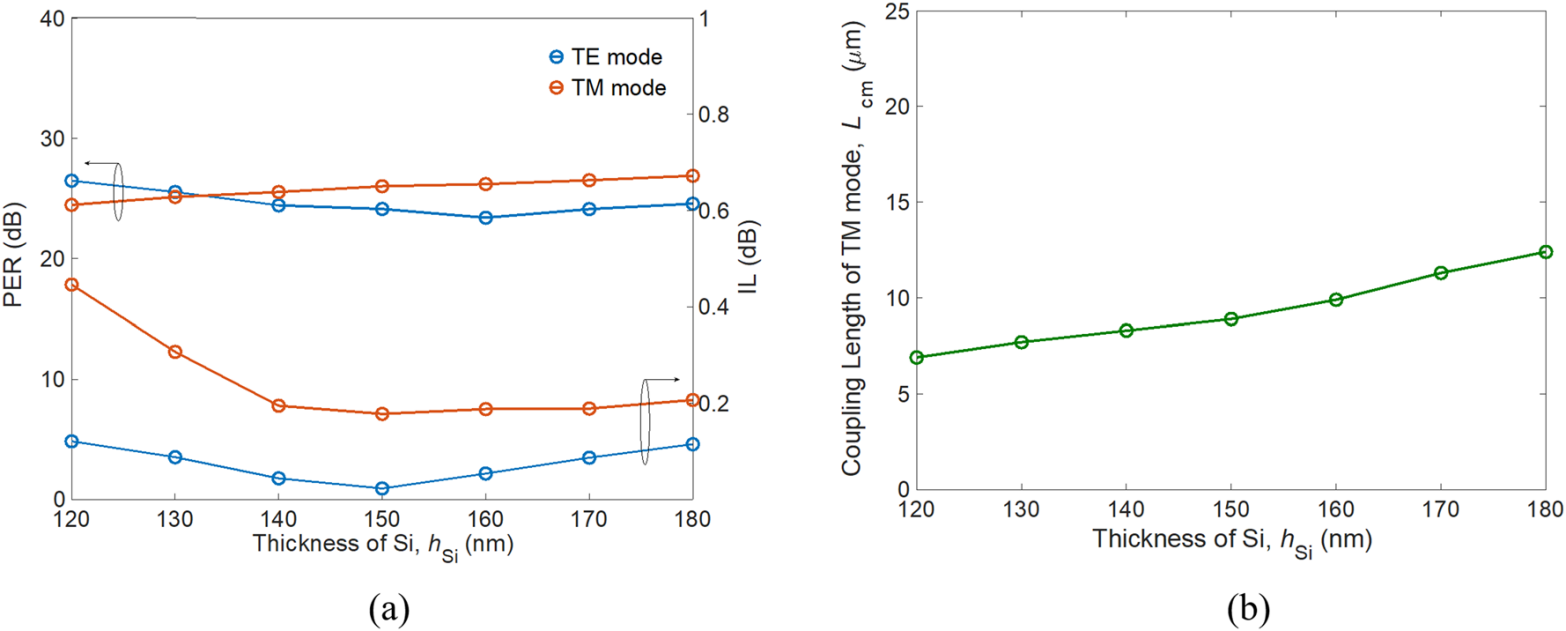
(**a**) PER (left axis) and IL (right axis), and (**b**) modified coupling length of TM mode *L*_cm_ as a function of Si thickness *h*_Si_.

To analyze the performance on device geometry, PERs and ILs versus *t*_slot_ are shown in Fig. 8a. It appears that the IL_TE_ is minimal and PER_TM_ is maximal at *t*_slot_ = 55 nm due to the maximum *L*_TE,_ as predicted in “Results and discussion”. Superior performance can be observed within the range *t*_slot_ = 40–70 nm. As mentioned above, a thicker *t*_slot_ leads to looser mode confinement, lengthening the coupling of the TM mode, *L*_TM_ (also *L*_cm_), as shown in Fig. 8b. At the values of *t*_slot_ = 10 and 55 nm, the longest *L*_cm_ = 14.5 μm and shortest *L*_cm_ = 7.6 μm, respectively, are obtained. The dependence of *h*_Si_ on PERs and ILs is shown in Fig. 9a at *t*_slot_ = 55 nm. Excepting IL_TM_, PER_TE_, PER_TM_, and IL_TE_ show slight variation over the range *h*_Si_ = 120 nm to 180 nm with *L*_cm_ = 6.9 to 12.4 μm, respectively, as shown in Fig. 9b.

**Figure 10:**
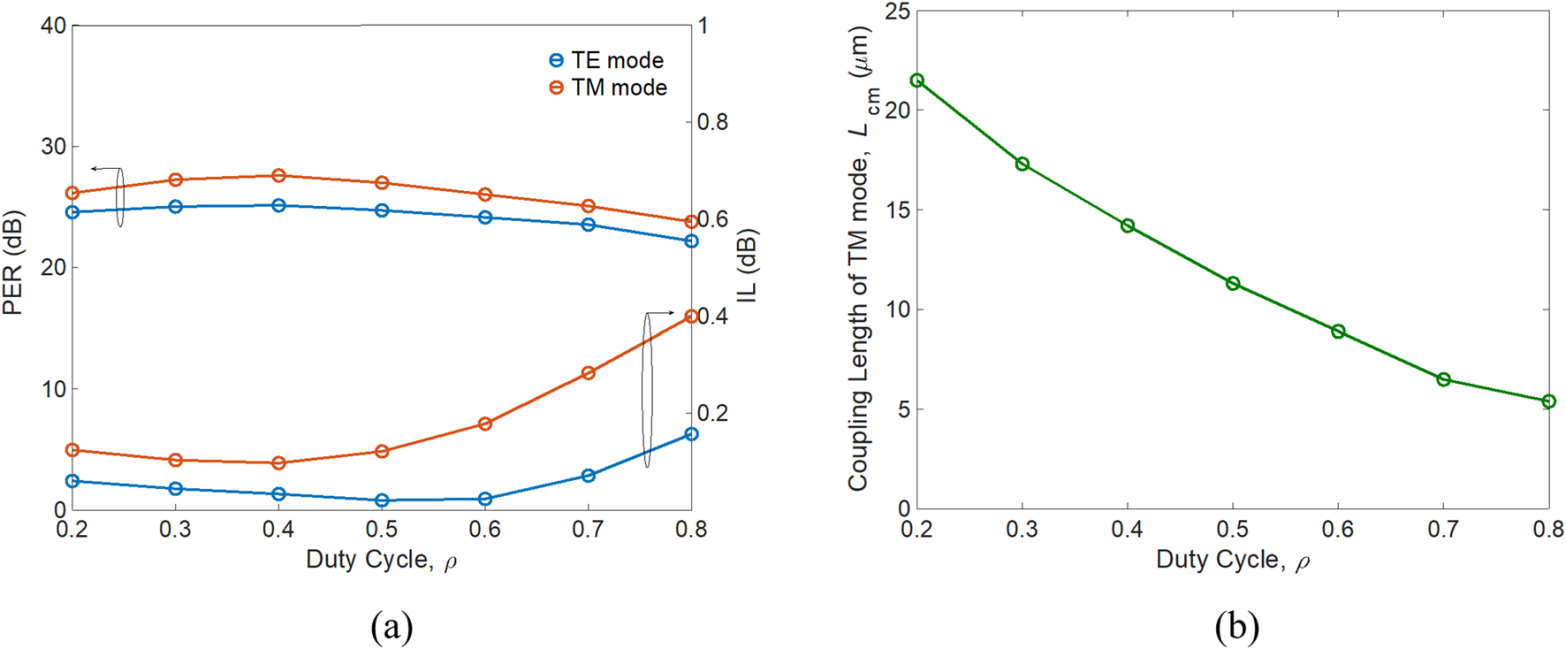
(**a**) PER (left axis) and IL (right axis), and (**b**) modified coupling length of TM mode *L*_cm_ as a function of duty cycle *ρ*.

As *h*_Si_ decreases, the TM mode shows looser confinement, resulting in higher IL_TM_. This is because a significantly higher ratio of TM power is distributed to the SiO_2_ substrate when *h*_Si_ is smaller than 140 nm. As a result, choosing the condition of *h*_*Si*_ > 150 nm preserves low TM mode loss. Considering the geometry variations of SWSWGs, PERs and ILs as a function of duty cycle *ρ* are shown in Fig. 10. After *ρ* > 0.5, the ILs of both modes increase significantly as *ρ* increases. This is attributed to weaker confinement of the TE and TM mode profiles. For the TE mode, this leads to more radiation loss while propagating through the curved waveguide. In contrast, greater power loss results from coupling of the TM mode into the cross bar. The *L*_cm_ significantly varies from 21.5 to 5.4 μm for *ρ* = 0.2 to *ρ* = 0.8, respectively, as shown in Fig. 10b. In experimental possibility, selecting a value close to *ρ* = 0.5 can effectively alleviate fabrication difficulties. Therefore, the trade-off between performance, footprint, and fabrication difficulty is to choose *ρ* = 0.6 with *L*_cm_ = 8.9 μm, rather than *ρ* = 0.5 with *L*_cm_ = 11.3 μm.

**Figure 11:**
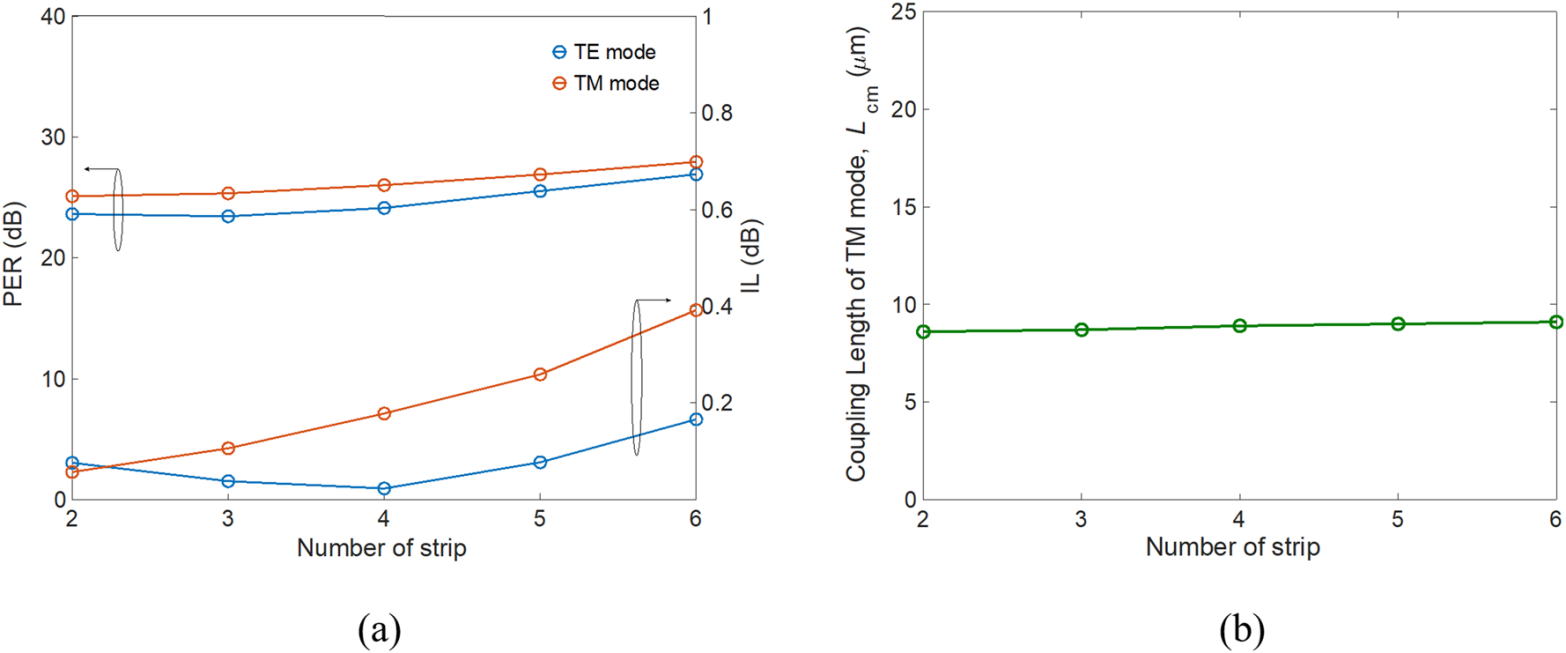
(**a**) PER (left axis) and IL (right axis), and (**b**) modified coupling length of TM mode *L*_cm_ as a function of number of strips.

In addition to the duty cycle, we also investigated PERs and ILs versus number of strips, as shown in Fig. 11.

**Figure 12:**
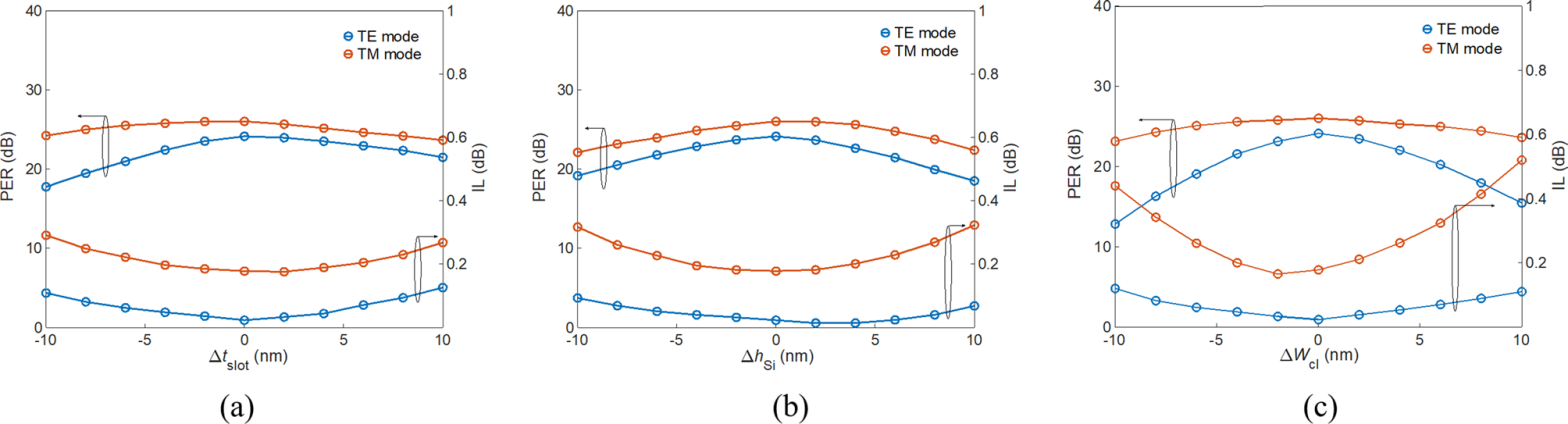
PER (left axis) and IL (right axis) as functions of variation in (**a**) slot thickness Δ*t*_slot_, (**b**) thickness of Si Δ*h*_Si_, and (**c**) width of SWSWGs Δ*W*_cl_.

It is known that the scattering loss increases as the number of strips increases, resulting in a significantly higher ILs. However, PERs increases moderately with the increase in number of strips. This is because although the loss of major power decreases for each bar, PER is dominated by the reduction of the other minor power, making the PER increase moderately. Notice that the device length is almost invariant with increasing number of strips. Finally, we evaluated fabrication tolerance of the proposed PBS. The PERs and ILs versus the variations of slot thickness, Δ*t*_slot_, thickness of Si layer, Δ*h*_Si_, and width of SWSWGs, Δ*W*_cl_ are shown in Fig. 12a–c, respectively. For Δ*t*_slot_ and Δ*h*_Si_, PER_TE_ and PER_TM_ achieve > 20 dB and IL_TE_, and IL_TM_ are < 0.3 dB for the variations in Δ*t*_slot_ and Δ*h*_Si_ within ± 10 nm. Thanks to the present experimental technology, the surface roughness of thin film depositions for SiO_2_ and Si layers is smaller than 5 nm using widely used plasma-enhanced chemical vapor deposition^34^.

For Δ*W*_cl_, PER_TE_ and IL_TM_ vary significantly because of the short *L*_TM_, resulting in a large deviation from the PMC. By contrast, PER_TM_ and IL_TE_ are slight variation due to the almost infinite long coupling length (achieving almost zero crosstalk) of the TE mode (*L*_TE_ = 151.3 mm at the transition point of *t*_slot_ = 55 nm, *W*_cl_ = 75 nm, and *h*_Si_ = 150 nm). For the condition of Δ*W*_cl_ < 5 nm, PER_TE_ > 20 dB, PER_TM_ > 25 dB, IL_TM_ < 0.06 dB, and IL_TM_ < 0.3 dB. From the calculated results, the most critical geometry affecting performance is the width of SWSWG. Fortunately, a mixed inductively coupled plasma-reactive ion etching process and hydrogen annealing^35^ can achieve sidewall roughness of a Si strip to < 1 nm.

In conclusion, we propose an ultra-compact, high-performance PBS, consisting of slotted waveguides with SWSWGs. By controlling the optical momentum of evanescent waves with the anisotropic SWSWGs, the coupling strength of the TE mode is suppressed remarkably, and that of TM mode is significantly enhanced. As a result, the proposed PBS significantly improves the PER of the TM mode by around 13 dB and reduces device length by > 70% (from 32.2 to 8.9 μm), when compared with a CSWC that does not include SWSWGs. Extraordinarily, the transition point of *n*_sym_ $\approx$ *n*_asym_ found in this work almost entirely eliminates TE mode crosstalk between the waveguides. This interesting phenomenon could be applied to building highly dense PICs and will be studied in depth elsewhere. In terms of practical fabrication, the required steps were identical to those of a CWSC, making fabrication of the proposed PBS comparatively simple. Our numerical simulations demonstrate that the proposed PBS achieves PER_TM_ > 25 dB, PER_TE_ > 20 dB, IL_TE_ < 0.05 dB, and IL_TM_ < 0.3 dB within a BW of ~ 50 nm from λ = 1,530 to 1,580 nm. In terms of fabrication tolerance, PER_TE_ and PER_TM_ achieve > 20 dB, and IL_TE_ and IL_TM_ are < 0.3 dB for variations in slot thickness and Si thickness within ± 10 nm. These calculated results show that the critical geometry is SWSWG width.

## Methods

According to the effective-medium theory (EMT)^31^, which limits the grating pitch *Λ* to a smaller than subwavelength scale, the SWG regions demonstrate equivalent material anisotropy as follows:

1 $$ \varepsilon_{EMT} = \left[ {\begin{array}{*{20}c} {\varepsilon_{x} } & 0 & 0 \\ 0 & {\varepsilon_{y} } & 0 \\ 0 & 0 & {\varepsilon_{z} } \\ \end{array} } \right], $$

2 $$ \varepsilon_{y} = \varepsilon_{z} = \rho {\kern 1pt} \varepsilon_{H} + \left( {1 - \rho } \right)\varepsilon_{L} , $$

3 $$ \varepsilon_{x}^{ - 1} = \rho \varepsilon_{H}^{ - 1} + \left( {1 - \rho } \right)\varepsilon_{L}^{ - 1} , $$

where *ε*_*x*_, *ε*_*y*_, and *ε*_*z*_ are the equivalent permittivities in the *x*-, *y*-, and *z*-directions, respectively, and *ε*_*H*_ (here *ε*_*Si*_) and *ε*_*L*_ (here *ε*_*air*_) are the high and low permittivities of the SWG. Additionally, the PER and IL of the two modes are defined in Eqs. (4) and (5), respectively^15^:

4 $$ {\text{PER}}_{TE(TM)} = 10\,\;\log_{10} \left( {\frac{{P_{TE(TM),\,thr(cross)} }}{{P_{TM(TE),\,thr(cross)} }}} \right), $$

5 $$ {\text{IL}}_{TE(TM)} = \; - 10\,\;\log_{10} \left( {\frac{{P_{TE(TM),\,thr(cross)} }}{{P_{input} }}} \right), $$

where *P*_*input*_ is the input power, *P*_*TE*(*TM*)*, thr*(*cross*)_ is the TE (TM) mode power at the through (cross) channel, and *P*_*TM*(*TE*)*, thr* (*cross*)_ is the TM (TE) mode power at the through (cross) channel.
